# Health-related quality of life among extrapulmonary tuberculosis patients and inequalities by disease manifestations: a longitudinal study analysing the impact of TB treatment

**DOI:** 10.1007/s11136-024-03860-4

**Published:** 2024-12-05

**Authors:** Shoaib Hassan, Manju Raj Purohit, Mala Kanthali, Reza Yaesoubi, Swapnil Jain, Tehmina Mustafa

**Affiliations:** 1 Centre for International Health, Department of Global Public Health and Primary Care, University of Bergen, Bergen, Norway; 2 Yale School of Public Health, Yale University, New Haven, USA; 3 Department of Pathology, R.D. Gardi Medical College, Ujjain, India; 4 Department of Public Health Sciences, Karolinska Institute, Stockholm, Sweden; 5 Department of Respiratory Medicine, R.D. Gardi Medical College, Ujjain, India; 6 Department of Thoracic Medicine, Haukeland University Hospital, Bergen, Norway

**Keywords:** Extrapulmonary Tuberculosis, EPTB, Quality of Life, HRQoL, PROMs, Inequalities

## Abstract

**Background:**

To understand the impact of extrapulmonary tuberculosis (EPTB) and its treatment on quality of life, we analysed patient-reported outcome measures (PROMs) among presumptive ETPB patients.

**Methods:**

EuroQol’s five-dimensional three-level (EQ-5D-3L) questionnaire and the Visual Analogue Scale (EQ-VAS) were used to measure PROMs by 274 presumptive EPTB patients at pre- and post-treatment stages. The patients were categorised as TB and non-TB by using a composite reference standard. Following the EuroQol suggested analysis methods, we calculated the health utility summary measure at the pre- and post-treatment stages. The health state density curve and index were used to analyse inequality in reported health profiles. We investigated factors associated with EPTB patients’ health utility through multivariable regression at the pre-treatment stage.

**Results:**

The analysis of PROMs showed both physical (mobility, self-care, usual activities) and psychological (pain, discomfort, anxiety & depression) health affected by all EPTB manifestations (lymphadenitis, pleuritis, meningitis and others). Moreover, we found inequality in reported health profiles across disease manifestations at pre- and post-treatment stages. Post-treatment, we found improvement in PROMs and no reports of extreme-level health problems. However, some problems persisted across all dimensions of EPTB manifestations. We found 100% improvement in pleuritis and meningitis manifestations. Socioeconomic status, type of health facility attended, and patients’ working capacity were associated with health utility.

**Conclusion:**

Despite post-treatment improvement in health, inequality of reported health states by EPTB manifestations persisted, though decreased. This highlights that holistic patient- and health system-level interventions addressing the impact of illness should consider EPTB by its manifestations, not as a single disease entity.

**Supplementary Information:**

The online version contains supplementary material available at 10.1007/s11136-024-03860-4.

## Abstract (plain language summary)

Extrapulmonary tuberculosis (EPTB) is a relatively less lethal disease than pulmonary tuberculosis. However, it carries other challenges, such as signs and symptoms that mimic other diseases, adding to its challenges in prompt diagnostic confirmation. Because ETPB is not readily transmissible to other patients and is less lethal, it has been ignored in public health policies and research. Eventually, to date, little to no information is available about its impact on patients’ quality of life. This study indicates that the known clinical manifestations of EPTB vary in their effect on health-related quality of life, including physical and psychosocial health. Similarly, despite successful completion of EPTB treatment, the maximum level of health was not achieved among all patients. Some patients still reported physical and psychosocial problems. This means that EPTB patients may still need support for their physical and psychosocial health after completing their medical treatment. Therefore, public health policy should also consider EPTB for its impact on quality of life. Moreover, EPTB should not be perceived as a single disease as its impact varies based on clinical manifestations.

**Supplementary Information:**

The online version contains supplementary material available at 10.1007/s11136-024-03860-4.

## Background

Tuberculosis (TB) remains a major global health issue, with the World Health Organization (WHO) reporting 10.6 million cases worldwide in 2021 [1]. The disease burden is higher in Southeast Asia and Africa [1, 2]. In low- and middle-income countries (LMICs), the burden of EPTB is exacerbated by limited healthcare resources, poor access to diagnostic tools, and delayed treatment initiation​ [2]​. High-income countries are not immune to these challenges, as EPTB often affects vulnerable populations such as immigrants, older adults, and those with comorbid conditions, leading to similar delays in diagnosis and treatment [1, 3, 4]​​. In contrast with pulmonary TB (PTB), which has been extensively studied, there is limited understanding of EPTB, affecting organs other than the lungs and its challenges that impact patients’ health-related quality of life (HRQoL) [5, 6]. EPTB constitutes a substantial portion of TB cases, yet it is often under-researched compared to PTB. The diverse manifestations of EPTB, including TB of the lymph nodes, bones, joints, and central nervous system, contribute to varied and severe health outcomes​ [6]​. These conditions can lead to prolonged illness, disability, and extensive treatment periods, all of which detrimentally affect patients’ physical, psychological, and social well-being​ [3, 6].

Although the available literature is limited, it shows that EPTB manifestations are associated with varying patterns of healthcare access, utilisation and treatment response, resulting in a spectrum of improvement in HRQoL per the patient-reported outcome measures (PROMs) [6]. The importance of researching HRQoL in patients with EPTB cannot be overstated. Studies have shown that TB, in general, significantly impacts HRQoL, reducing physical health and mental well-being [3, 6–9]. However, most research has focused on PTB, leaving a gap in understanding the full scope of HRQoL impairments in EPTB patients [10]. Understanding this gap is critical so that the estimation of the impact of EPTB on patients’ health and the factors influencing it can be quantified. Recent systematic reviews and meta-analyses have highlighted the severe HRQoL impairments in TB patients [8]. However, a lack of dedicated analysis still underscores the need for studies addressing HRQoL in EPTB cohorts​.

Unless such estimations based on PROMs are made for EPTB patients, we cannot ascertain how various dimensions of patients’ health are affected by this illness. The PROMs are also advantageous in diseases with a broad spectrum of symptoms, such as extrapulmonary tuberculosis. They can capture the patient’s subjective experience of disease, allowing for a more holistic assessment of health impacts, including physical, emotional, and social dimensions [6]. Research has shown that PROMs are better at detecting symptoms which patients can better assess [11]. For example, clinicians cannot estimate the level of pain, fatigue, and mental health issues, which the patients may better report. Therefore, in conditions like extrapulmonary TB, where symptoms can vary widely, PROMs are valuable in capturing EPTB’s true impact across various health dimensions. To our knowledge, only one study from Zanzibar has reported PROMs of EPTB [6]. Moreover, understanding the HRQoL based on PROMs in our study was deemed imperative for several reasons [6, 9, 12]. Firstly, enhanced HRQoL assessments addressing patients’ physical and psychological aspects of health can lead to more holistic treatment approaches​. Secondly, data on HRQoL in EPTB can inform public health policies and clinical guidelines, ensuring they are tailored to the specific needs of the affected population. Thirdly, identifying key areas where EPTB patients suffer the most can help strategically allocate resources, improving healthcare delivery and patient outcomes​. Fourthly, by improving the quality of life for EPTB patients, healthcare systems can potentially reduce the overall burden of TB, as better-managed patients are less likely to suffer from severe long-term consequences or require extensive additional care. Finally, patients’ reported information about their HRQoL using the internationally adopted measuring instruments can support a comparison of bearings across diseases. In conclusion, research focused on EPTB patients’ HRQoL is crucial for understating the impact of TB treatment and care, particularly in high-burden regions.

This study aims to bridge the knowledge gap by analysing EPTB PROMs based on EuroQol’s five-dimensional three-level (EQ-5D-3L) questionnaire [12]. To highlight the impact of EPTB on patients’ health, we compare their HRQoL with that of non-TB patients at the pre-treatment stage and analyse the post-treatment changes in HRQoL of EPTB patients. We also aimed to identify factors associated with EPTB patients’ reported health utilities categorised by disease manifestations at the pre-treatment stage.

## Methods

### Study participants and data collection

This institution-based prospective cohort study, which lasted two years, from April 2018 to February 2020, was based at the Chandrikaben Rashimikant Gardi Hospital (CRGH), which is associated with Ruxmaniben Deepchand Gardi Medical College (RDGMC) in Ujjain, Madhya Pradesh, India. Throughout the study, presumed EPTB patients examined by clinicians were enrolled from the inpatient and outpatient departments. Patients who had received anti-tuberculous treatment (ATT) during the last 12 months or did not provide informed consent were excluded from the study. The sample size for this study was calculated based on the findings from previous studies. Keeping a view of findings from earlier studies [13–16], with a statistical significance level of 0.05 and a power of 0.90, the total required sample size was estimated at 200. However, patient enrolment was planned to continue throughout the study’s duration. We followed the Strengthening the Reporting of Observational Studies in Epidemiology (STROBE) guidelines for cohort studies (Online Resource 1) [17].

Using the pre-designed questionnaire, two trained hospital staff interviewed the enrolled patients to collect demographic, socioeconomic, medical history, and information about the current illness. Subject matter experts evaluated the content validity of the questionnaire. A similar questionnaire has also been used in our two study sites [6, 18–20]. After enrolment, we offered investigations and treatment to presumptive EPTB patients per the Indian National Tuberculosis Control Programme guidelines. Qualified medical doctors conducted the relevant clinical examination and recorded their findings. To classify patients based on their EPTB or non-TB status, we utilised the composite reference standard (CRS), which is well-established for presumptive EPTB patients and has been applied in this Indian study setting and Tanzania, whose details are published elsewhere [6, 18, 21–23]. According to the CRS, individuals not identified as EPTB-positive were categorised as non-TB patients. The presumptive EPTB patients were diagnosed using laboratory tests and clinical criteria, details of which have been published elsewhere. After the initiation of ATT as an intervention in this study, EPTB patients were followed for a maximum of one year to monitor disease progression and treatment effectiveness. Patients not starting ATT were followed until recovery or a diagnosis other than TB.

As an outcome measure in this study, the HRQoL of the presumptive EPTB patients was assessed using EuroQol’s five-dimensional three levels (EQ-5D-3L) questionnaire and the Visual Analogue Scale (EQ-VAS), which has an established feasibility and reliability in TB populations [3, 8, 24]. The EQ-5D-3L questionnaire described five dimensions: mobility, self-care, usual activity, pain/discomfort, and anxiety/depression. Each dimension had three levels: level 1: no problem, level 2: some problem, and level 3: extreme problem. The EQ-VAS was a visual analogue scale from 0 to 100. The study participants reported the perception of their current health state, with 0 being the worst imaginable health state and 100 being the best imaginable health state.

A specialist doctor participating in the study also randomly joined interviews to ensure interview adherence to set standards. Although patients of all age groups were enrolled, this study analysis included adult patients because, per the study implementation, we had HRQoL data collected only from the adult patients. We ensured patients’ privacy during the interview process. We transferred all the information from the interviews to the electronic version produced in Microsoft Excel, and each patient received a unique identifier.

### Statistical analysis

We performed a descriptive analysis of demographic, socioeconomic and clinical information. Comparisons of proportion were done by using the Chi-square test. A p-value < 0.05 was considered statistically significant. Then, we analysed the PROMs per the EuroQol group’s suggested methods, published elsewhere [14, 15], while a brief description follows.

(i) **Cross-sectional analysis**: Per the EuroQol group’s recommendations [25], we first conducted a cross-sectional study analysis by calculating the frequencies of PROMs for EPTB and non-TB patients at the pre- and post-treatment stages to estimate the impact of illness on the five dimensions of health. This analysis included quantifying the cumulative frequencies of the most frequently reported health profiles. Moreover, we summarised health profiles by subgroups of EPTB manifestations at pre- and post-treatment stages.
(ii) **Longitudinal analysis**: To describe the impact of treatment, we conducted a longitudinal study analysis of PROMs at the pre- and post-treatment stages.
  - We estimated changes in the health profile data using the Paretian Classification of Health Change (PCHC) [26]. This approach is based on the principles of Pareto improvement in Welfare Economics and allows summarising health changes in ordinal terms (improve, worsen or no change).
  - We measured non-parametric effect size as the probability of superiority (PS) to estimate changes in health (improvement or deterioration) [25]. The effect size PS refers to the likelihood that when selecting a pair of observations from two groups, the observation from the second group will be greater on one occasion than from the second [27]. It is a recommended method by the EuroQol Group, and we deemed it suitable to illustrate the difference between the PROMs data for various forms of EPTB manifestations on two occasions [14, 15]. The PS estimates ranged from 0 to 1 (< 0.5 if more patients deteriorate than improve, equal 0.5 if the same number of patients improve and deteriorate or do not change and > 0.5 if more patients improve than deteriorate.
  - We summarised the ordinal health profile PCHC as the Health Profile Grid (HPG) [14]. The x-axis and y-axis show health profiles (1 to 243) ordered from best to worst health. Each point on the grid shows improvement, worsening, or no change in health according to the rank order across the 45^o^ line, which represents no change. The ranks above the line represent improvement, while demarcate worsening below the line.
  - The distribution of health profile data can be represented by a Health State Density Curve (HSDC) to illustrate its evenness. The distribution of health profiles in the HSDC is ranked from the most to the least frequent profile, with ties in frequency being ranked arbitrarily. The cumulative distribution of the total distinct health profiles is represented on the Y-axis. Meanwhile, the X-axis shows the cumulative distribution of patients [28]. We plotted a graphical distribution of health profiles pre- and post-treatment as the HSDC. This graph is analogous to the Lorenz curve that describes the concentration of income distribution. In comparison, the 45^o^ line shows an equal spread of health profiles. A concentrated distribution would depict fewer health profiles reported by a higher proportion of patients. Thus, the HSDC would be farther from the diagonal line as the distribution of PROMs got more uneven (a larger proportion of the study population commonly reports relatively fewer health profiles, corresponding to the x-axis and y-axis of the HSDC).
  - We approximated inequality by estimating the Health State Density Index (HSDI), the area between HSDC and the 45^o^ line [28]. The HSDI corresponds to the Gini coefficient that accompanies the Lorenz curve. Its value ranges from 0 to 1, presenting total inequality and equality. This measure would allow comparing inequality across PROMs for different instruments or diseases.
(iii) For the VAS data, we estimated minimum and maximum values and median with IQR in various EPTB disease manifestations at the pre-treatment and post-treatment stages.

Kendall’s rank correlation test was applied to determine the correlation between EQ-5D-3L and the VAS. We estimated Kendall’s rank correlation coefficient (Kendall’s tau) to assess whether there is a rank-based association. The tau ranges between − 1 and 1, indicating a reverse and same-order correlation.

The EQ-5D-3L consists of five dimensions: Mobility, Self-care, Usual Activities, Pain/Discomfort, and Anxiety/Depression, each with three response levels ranging from no problems to extreme problems (rated 1–3). These levels can be combined to define 243 potential health states, from 11,111 (perfect health) to 33,333 (worst health). Each health state is associated with a single “utility” score based on these EQ-5D-3L profiles. Finally, based on these PROMs per the EQ-5D-3L, we summarised health profiles into a single summary measure called health utility. A health utility value is estimated using country-specific societal preference weights (or value sets) and applied to the PROMs. For example, a health utility estimate of 1 would have a PROM of 1 on all five dimensions (11111) of patient-reported health profiles and refers to a maximum possible health state. A health utility estimate of 0 means dead. If a patient reports 2 or 3 for any dimension of the EQ-5D-3L questionnaire, a specific value corresponding to either 2 or 3 would be subtracted from 1 (the maximum possible health level). The health utility estimates also allow negative values to be assigned to PROMs deemed worse than dead. Because the country-specific EQ-5D-3L value set was unavailable for India, we used the value set based on neighbouring Pakistan [29]. Additional details about the value sets used to estimate health utility from the patient-reported health profile and methods as recommended by the EuroQol group are documented in other publications [30, 31].

We used univariable regression to study potential factors associated with EPTB patients’ health utilities. Factors that had a significant association (*p* < 0.05) during the univariable regression analysis were considered for the stepwise multivariable regression model [32]. We reported the final multivariable model with statistically significant (p-value < 0.05) coefficients and 95% confidence intervals (CI). We conducted all statistical analyses using R software employing the ggplot, tidyverse and Eq5d packages [33–35].

In addition to the EPTB-focused analysis described above, we also compared EPTB PROMs with non-TB patients to assess the impact of EPTB on HRQoL, especially at the pre-treatment stage. A systematic record of treatment and alternate diagnosis of non-TB patients was not available. However, we had information about their PROMs per the EQ5D questionnaire, as most non-TB patients were also followed up like EPTB patients.

## Results

During the study period, we registered 463 presumptive EPTB patients (Online Resource 2). In addition to the patients lost from follow-up (*n* = 10), we excluded 39 patients on TB treatment during the past 12 months (which was one of the exclusion criteria). Moreover, two patients were excluded due to not providing informed consent or agreeing to the interview. We did not have information about PROMs from children (under 16 years old). Therefore, 56 children are not included in this study analysis. Of the remaining 356 presumptive EPTB patients, we included patients whose PROMs data was available. We did not have enough data points to impute the PROMs of these missing data. A total of 274 adult presumptive EPTB patients (178 EPTB and 96 non-TB patients classified per the CRS, Online Resource 2) were included in this study’s final analysis of PROMs. The average age of EPTB and non-TB patients was 31 and 37 years, respectively. The analysis of demographic, socioeconomic and clinical factors showed that compared to non-TB patients, EPTB patients reported a higher proportion (p-value < 0.05) of females, 16–29 years old, middle-high socioeconomic status, family history of TB, patient delay, health system delay, duration of weight loss & night sweats, > 60 min of travel & weight time to the health facility (HF) providing diagnosis (study site) and > 25% reduced working capacity (Table 1). EPTB patients had varying disease manifestations: lymphadenitis (69%), pleuritis (22%), meningitis (6%) and others (3%, including ascites (*n* = 4) and osteomyelitis (*n* = 2)).

### Cross-sectional analysis: impact of EPTB manifestations on HRQoL

Of the 243 possible health states per the EQ-5D-3L (ranging from best to worst health state: 11111 to 33333), we found that compared to other EPTB manifestations, lymphadenitis EPTB patients’ HRQoL spread across the highest number of health states, 16 and 7 during the pre- and post-treatment stages, respectively (Online Resource 3). This was followed by patients manifesting pleuritis, meningitis and others (Table 1). Patients reporting health states as no problem across all dimensions (11111) at the pre-treatment stage were highest among lymphadenitis (52%), followed by meningitis (9%) and pleuritis (5%) manifestations. While, the non-TB patients reporting no problem across dimensions were only among lymphadenitis (72%).

**Table 1:** Presumptive extrapulmonary tuberculosis (EPTB) patients by demographic, socioeconomic and clinical factors

Factors associated with pre-treatment health utility		EPTB patient	Non-TB patients	*p*-value*
Gender	Female	107 (60.1)	45 (46.9)	0.048
	Male	71 (39.9)	51 (53.1)	
Age-groups (years)	16–29	103 (57.9)	38 (39.6)	0.006
	30–44	42 (23.6)	26 (27.1)	
	Above 45	33 (18.5)	32 (33.3)	
Education levels	Primary or below	104 (58.4)	56 (58.3)	0.923
	Middle or Secondary	51 (28.7)	29 (30.2)	
	Higher	23 (12.9)	11 (11.5)	
Marital status	Married	119 (67.2)	66 (68.8)	0.904
	Unmarried	58 (32.8)	30 (31.2)	
Socioeconomic status (SES)	Low SES	26 (14.6)	27 (28.1)	0.010
	Middle SES	68 (38.2)	38 (39.6)	
	High SES	84 (47.2)	31 (32.3)	
Occupation	Govt Employed	31 (17.4)	19 (19.8)	0.785
	Housewife	63 (35.4)	30 (31.2)	
	Unemployed	41 (23.0)	26 (27.1)	
	Private Employed	43 (24.2)	21 (21.9)	
Previous history of TB	No	157 (88.2)	88 (91.7)	0.494
	Yes	21 (11.8)	8 (8.3)	
Family history of TB	No	145 (81.5)	89 (92.7)	0.019
	Yes	33 (18.5)	7 (7.3)	
Self-medication	No	144 (80.9)	81 (84.4)	0.582
	Yes	34 (19.1)	15 (15.6)	
Hospitalisation status	Inpatient	74 (41.6)	32 (33.3)	0.228
	Outpatient	104 (58.4)	64 (66.7)	
Patient-level delay	Above Median PD	83 (46.6)	31 (32.3)	0.030
	Below Median PD	95 (53.4)	65 (67.7)	
Health system level delay (before study site visit)	Above Median SD	96 (53.9)	37 (38.9)	0.026
	Below Median SD	82 (46.1)	58 (61.1)	
Health system level delay (after study site visit)	Below Median SD	106 (59.6)	45 (46.9)	0.059
	Above Median SD	72 (40.4)	51 (53.1)	
Duration of fever	Mean (SD)	36.9 (93.8)	14.7 (30.2)	0.025
Duration of weight loss	Mean (SD)	43.9 (84.2)	37.4 (117.9)	0.596
Duration of appetite loss	Mean (SD)	39.0 (84.3)	25.6 (93.6)	0.229
Duration of night sweats	Mean (SD)	21.0 (68.8)	6.7 (22.8)	0.049
Duration of fatigue	Mean (SD)	32.9 (79.5)	25.2 (87.8)	0.467
Duration of amenorrhea	Mean (SD)	9.5 (46.9)	5.0 (20.8)	0.530
Duration of body weakness	Mean (SD)	60.4 (157.7)	30.2 (86.9)	0.083
Duration of frequent colds	Mean (SD)	12.3 (46.8)	5.7 (21.5)	0.190
Duration of neck mass	Mean (SD)	54.1 (135.0)	69.2 (191.6)	0.450
Number of health facilities visited for this illness	One	63 (35.4)	23 (24.0)	0.088
	Two	62 (34.8)	32 (33.3)	
	Three	9 (5.1)	4 (4.2)	
	Four	0 (0.0)	1 (1.0)	
	Missing	44 (24.7)	36 (37.5)	
Number of visits to health facilities for this illness	One	52 (29.2)	22 (22.9)	0.419
	Two	69 (38.8)	32 (33.3)	
	Three	14 (7.9)	10 (10.4)	
	Four	5 (2.8)	3 (3.1)	
	Missing	38 (21.3)	29 (30.2)	
Time to reach the nearest health facility	Below 30 min	67 (37.6)	48 (50.0)	0.039
	Between 30–60 min	68 (38.2)	36 (37.5)	
	Above 60 min	43 (24.2)	12 (12.5)	
Time to reach this health facility (study site)	30–60 min	47 (26.4)	18 (18.8)	0.347
	Above 60 min	43 (24.2)	24 (25.0)	
	Below 30 min	88 (49.4)	54 (56.2)	
Travel and wait time to this health facility (study site)	Below 10 min	100 (56.2)	63 (65.6)	0.039
	10–60 min	32 (18.0)	22 (22.9)	
	60–120 min	32 (18.0)	9 (9.4)	
	Above 120 min	14 (7.9)	2 (2.1)	
Health facilities visited for this illness	Dispensary	86 (48.3)	52 (54.2)	0.699
	District Hospital	17 (9.6)	6 (6.2)	
	Health Center/Others	5 (2.8)	1 (1.0)	
	Private Hospital	3 (1.7)	2 (2.1)	
	Regional Hospital	67 (37.6)	35 (36.5)	
Duration of reduced working capacity	Below 15 days	84 (47.2)	46 (48.4)	0.600
	16–30 days	58 (32.6)	26 (27.4)	
	Above 30 days	36 (20.2)	23 (24.2)	
Percentage of reduced working capacity	Below 25%	16 (9.0)	31 (32.3)	< 0.001
	25–50%	87 (48.9)	44 (45.8)	
	51–75%	39 (21.9)	8 (8.3)	
	76–100%	36 (20.2)	13 (13.5)	

* p-value corresponds to the use of the Pearson chi-square and Kruskal Wallis tests

**Fig. 1:**
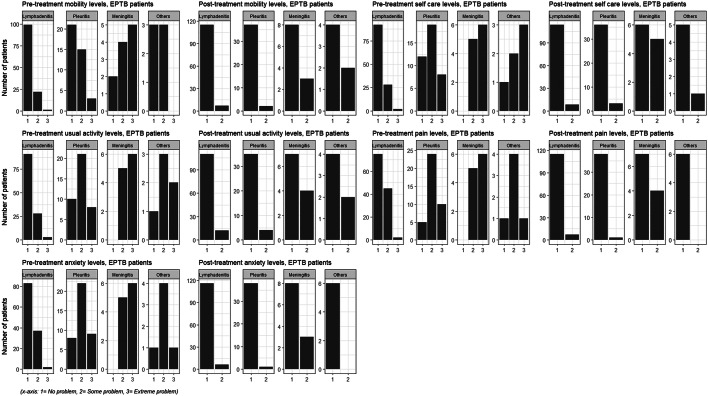
Frequency of EQ-5D-3L health states reported by extrapulmonary tuberculosis (EPTB) patients

In the pre-treatment phase, the impact of EPTB on HRQoL was evident across three levels of the five dimensions. Moreover, we found that a proportion of EPTB patients from all the disease manifestations reported their HRQoL affected to level three across five dimensions at the pre-treatment stage and none at the post-treatment stage (Online Resource 4). At the pre-treatment stage, most meningitis EPTB patients (> 50%) reported the highest impact (extreme problem) of illness across the five dimensions (Fig. 1). Level 3 health states reporting extreme problems across the five dimensions were less common among EPTB patients with pleuritis (5–10%) and lymphadenitis (1%).

**Fig. 2:**
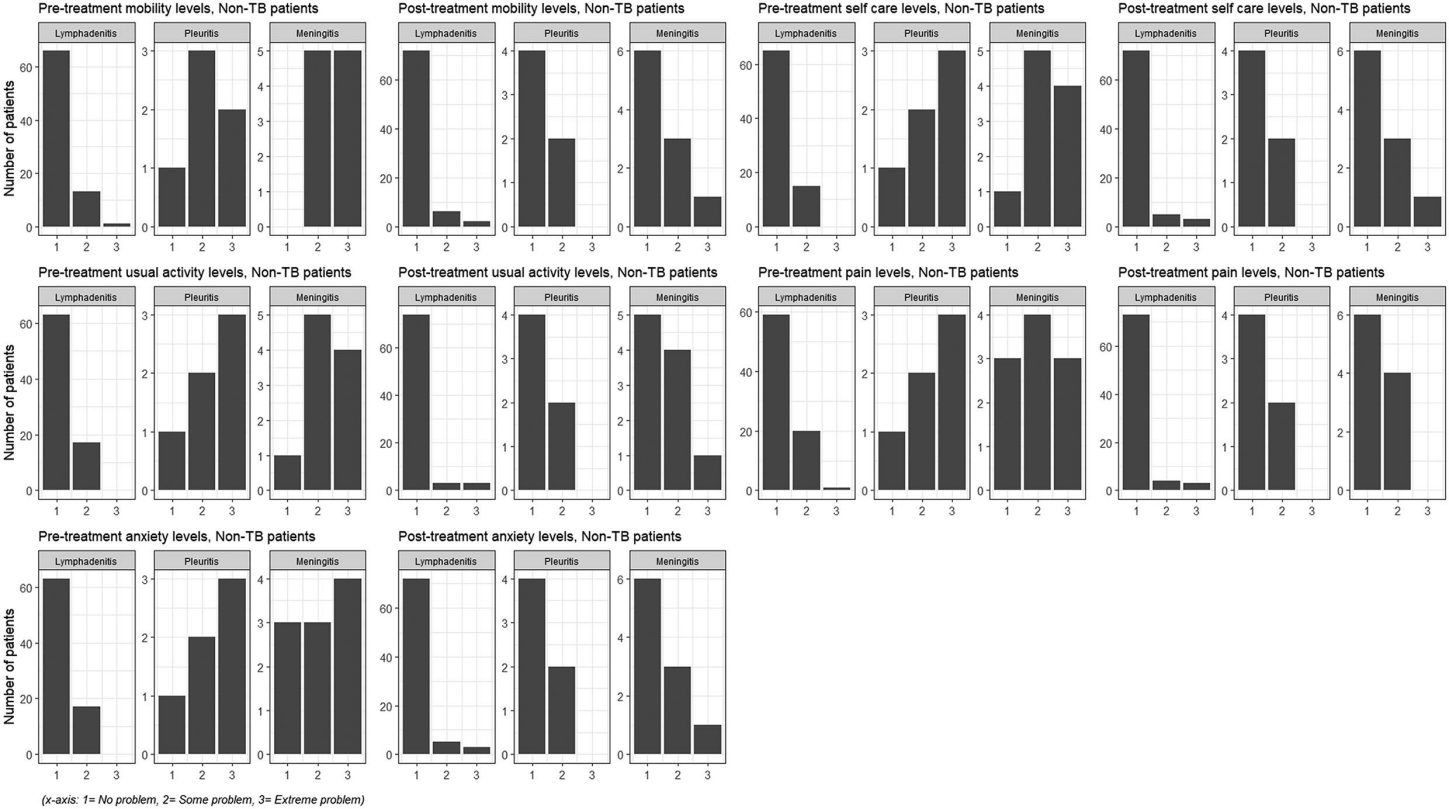
Frequency of EQ-5D-3L health states reported by non-tuberculosis (TB) patients

We did not have detailed information about non-TB diagnosis who presented as presumptive EPTB patients with a range of disease manifestations: lymphadenitis (83%), meningitis (11%) and pleuritis (6%). Of whom, 49% reportedly recovered without treatment or received other diagnoses: tumours (5%, including benign and malignant) and thyroid-related conditions (5%). Non-TB patients also reported extreme-level problems in their health states at the post-treatment follow-up stage (Fig. 2).

### Longitudinal analysis: impact of treatment on HRQoL

At the post-treatment stage, using the PCHC of EPTB patients, we found a 100% improvement in PROMs among all disease manifestations except lymphadenitis. We found that 5% of EPTB patients with lymphadenitis had no change or worsening in their HRQoL (Table 2). Among the three lymphadenitis patients who reported worsening HRQoL, they had the maximum possible health states (11111) across all dimensions at the pre-treatment stage. which decreased to 11211, 22211 and 22222. About 23% of non-TB patients manifesting lymphadenitis also reported worsened HRQoL.

**Table 2:** Health states reported by Extrapulmonary Tuberculosis (EPTB) and non-TB patients and changes following TB treatment

	EPTB patients		Non-TB patients	
	Number (%)		Number	
Lymphadenitis	No change	3 (5)	No change	0 (0)
	Improve	56 (90)	Improve	20 (77)
	Worsen	3 (5)	Worsen	6 (23)
	Mixed change	0 (0)	Mixed change	0 (0)
	Total with problems	62 (51)	Total with problems	26 (33)
	No problems	60 (49)	No problems	54 (67)
Meningitis	No change	0 (0)	No change	1 (10)
	Improve	10 (100)	Improve	9 (90)
	Worsen	0 (0)	Worsen	0 (0)
	Mixed change	0 (0)	Mixed change	0 (0)
	Total with problems	10 (91)	Total with problems	10 (100)
	No problems	1 (9.1)	No problems	0 (0)
Pleuritis	No change	0 (0)	No change	0 (0)
	Improve	37 (100)	Improve	6 (100)
	Worsen	0 (0)	Worsen	0 (0)
	Mixed change	0 (0)	Mixed change	0 (0)
	Total with problems	37 (95)	Total with problems	6 (100)
	No problems	2 (5)	No problems	0 (0)
Others	No change	0 (0)		
	Improve	5 (100)		
	Worsen	0 (0)		
	Mixed change	0 (0)		
	Total with problems	5 (83)		
	No problems	1 (17)		

While analysing the changes in post-treatment HRQoL by employing PS, we found that more patients improved (PS > 0.5) than deteriorated across all the EPTB manifestations. This change in HRQoL was highest for meningitis, followed by pleuritis and lymphadenitis (Table 3). Moreover, the improvement among all EPTB manifestations was noted across dimensions: physical (mobility, self-care, usual activities) and psychological (pain & discomfort and anxiety & depression). A similar improvement was also apparent for the non-TB patients manifesting pleuritis followed by meningitis and lymphadenitis (Table 3).

**Table 3:** Probability of superiority (PS) in change of health states at patients’ follow-up

	EPTB patients				Non-TB patients		
	Lymphadenitis	Pleuritis	Meningitis	Others	Lymphadenitis	Pleuritis	Meningitis
Mobility	0.57	0.72	0.86	0.58	0.53	0.92	0.85
Self-care	0.60	0.73	0.86	0.75	0.54	1	0.85
Usual activity	0.58	0.81	0.91	0.75	0.56	1	0.80
Pain & Discomfort	0.67	0.95	0.91	0.83	0.58	1	0.90
Anxiety & Depression	0.64	0.91	0.91	0.92	0.58	0.92	0.90

**Fig. 3:**
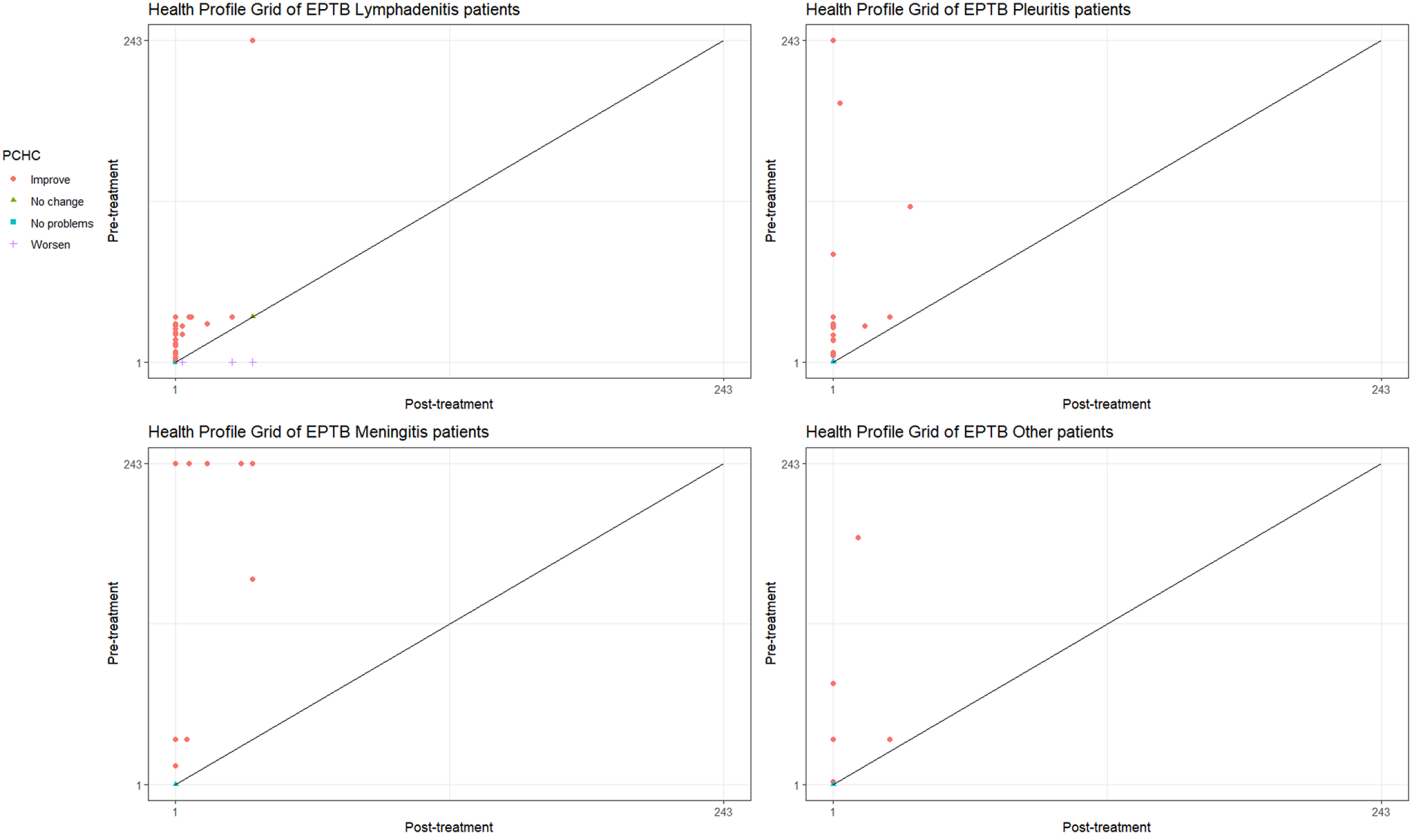
EPTB patients’ Paretian Classification of Health Change (PCHC) shown by Health Profile Grid (HPG)

Based on the PCHC plot in the HPG, we noted the EPTB illness was less severe for lymphadenitis, followed by pleuritis and meningitis (Fig. 3). Non-TB patients, too, showed similar patterns across the disease manifestations (Online Resource 5). Moreover, the HSDC plot showed an inequality in the distribution of reported health profiles.

**Fig. 4:**
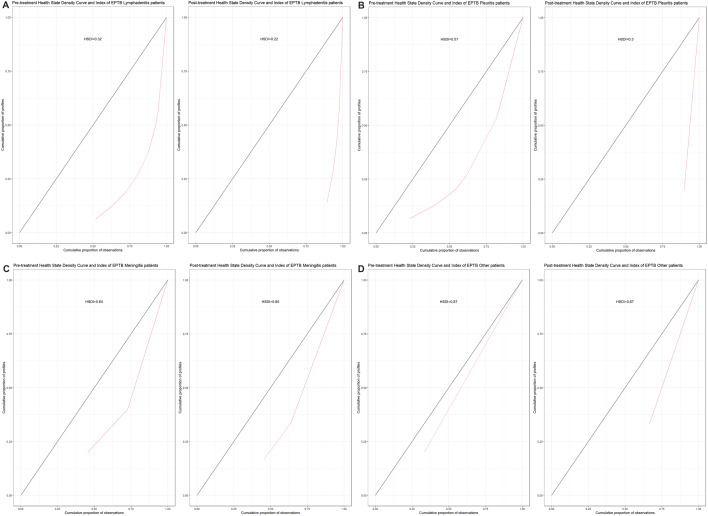
**A** EPTB lymphadenitis patients’ Health State Density Curve (HSDC) and Health State Density Index (HSDI). **B** EPTB Pleuritis patients’ Health State Density Curve (HSDC) and Health State Density Index (HSDI). **C** EPTB Meningitis patients’ Health State Density Curve (HSDC) and Health State Density Index (HSDI). **D** EPTB Other patients’ Health State Density Curve (HSDC) and Health State Density Index (HSDI)

We found that health profiles for lymphadenitis manifestations were most uneven at the pre- and post-treatment stages (HSDC further from the 45^o^ line), followed by pleuritis, meningitis and others (Fig. 4a-d). At the post-treatment stage, the HSDI value decreased for all EPTB manifestations except meningitis. The post-treatment decreases in HSDI (from 1 to 0), except for meningitis, meant an increase in inequality in HRQoL and was in line with the inequality shown by the HSDCs (Fig. 4a-d). However, neither the HSDC nor the HSDI can inform which health profiles are most reported as part of the PROMs. Therefore, analysing the HSDC and HSDI, along with the frequency of reported health profiles at pre- and post-treatment stages (described earlier as the cumulative frequency of health states), showed that a post-treatment increase in inequality is due to the higher concentration of health profiles corresponding to improved HRQoL. Thus, a larger proportion of EPTB patients’ HRQoL was reported by the fewer health profiles (of improved health). However, despite treatment, not all the EPTB patients achieved the maximum possible level of health (Tables 1, 2 and 3; Fig. 4a-d).

We estimated health utility as the single summary measure of the PROMs per the EQ-5D-3L questionnaire. This analysis showed the highest median score at the pre-treatment stage for lymphadenitis (1.00, IQR = 0.90- 1.00), followed by pleuritis (0.83, IQR = 0.78–0.93) and meningitis (0.46, IQR= -0.17- 0.78) (Table 4). At the pre-treatment stage, we also found reports of below 0 (worse than dead) estimates among EPTB patients manifesting lymphadenitis (*n* = 1), pleuritis (*n* = 2) and meningitis (*n* = 5). At the post-treatment stage too, a similar pattern of high median health utility was found across EPTB manifestations: lymphadenitis (1.00, IQR = 1.00–1.00), pleuritis (1.00, IQR = 1.00–1.00) and meningitis (0.94, IQR = 0.86- 1.00). None of the health utility estimates were below 0 at the post-treatment stage.

The VAS data showed improvement in health across all the EPTB manifestations (p-value < 0.05). Lymphadenitis manifestation had the most dispersed VAS, followed by pleuritis and meningitis. During the pre-treatment stages, the median VAS value was highest for lymphadenitis (50, IQR = 45–60), followed by pleuritis (40, IQR = 30–50) and meningitis (30, IQR = 15–45) (Table 4). At the post-treatment stage, the reported median VAS increased across all EPTB manifestations: lymphadenitis (80, IQR = 70–85), pleuritis (70, IQR = 65–80) and meningitis (60, IQR = 50–68).

**Table 4:** Summary of health utility and visual analogue scale (VAS) score reported by the Extrapulmonary Tuberculosis patients

	Health Utility, pre-treatment stage	Health Utility, post-treatment stage
	Min.1st Qu. Median 3rd Qu. Max.	Min. 1st Qu. Median 3rd Qu. Max.
Lymphadenitis	-0.17 0.90 1.00 1.00 1.00	0.78 1.00 1.00 1.00 1.00
Pleuritis	-0.17 0.78 0.83 0.93 1.00	0.78 1.00 1.00 1.00 1.00
Meningitis	-0.17 -0.17 0.46 0.78 1.00	0.78 0.86 0.94 1.00 1.00
Others	0.38 0.70 0.78 0.92 1.00	0.85 0.93 1.00 1.00 1.00
	**VAS**,** pre-treatment stage**	**VAS**,** post-treatment stage**
	Min. 1st Qu. Median 3rd Qu. Max.	Min. 1st Qu. Median 3rd Qu. Max.
Lymphadenitis	20 45 50 60 85	40 70 80 85 95
Pleuritis	10 30 40 50 65	35 65 70 80 90
Meningitis	05 15 30 40 45	45 50 60 68 85
Others	25 28 38 48 80	55 60 70 84 90

Min = Minimum, Max = Maximum, Qu = Quintile

At pre-treatment, Kendall’s tau statistic showed that the health profiles per the EQ-5D-3L and VAS are not highly correlated. The tau statistics (*p* < 0.05) varied by EPTB manifestation: lymphadenitis (-0.41), pleuritis (0.03) and meningitis (0.18). At the post-treatment stage, the tau statistics were as follows: lymphadenitis (-0.41), pleuritis (-0.43) and meningitis (1.0).

### Multivariable regression model

We conducted a regression analysis to understand the association of the health utility of EPTB patients at the pre-treatment stage with the demographic, socioeconomic and clinical factors (Online Resource 6). We identified factors significantly associated with lymphadenitis and pleuritis manifestations (Table 5). The final models showed that among lymphadenitis EPTB patients, health utility decreased with the increasing age (above 45 years old compared to 16–29 years old) (Coefficient − 0.10, 95% CI -0.16- -0.04), attending health centres as the first HFs for the ongoing EPTB illness (compared to dispensaries) (Coefficient − 0.23, 95% CI -0.34- -0.12), and reduced working capacity at 51–75% & 76–100% (compared to below 25%) (Coefficient − 0.08, 95% CI -0.15- -0.00 & Coefficient − 0.22, 95% CI -0.31- -0.12). The health utility for pleuritis EPTB patients was associated with an increase for middle and high SES (compared to low SES) (Coefficient 0.69, 95% CI 0.46–0.92 & Coefficient 0.73, 95% CI 0.52–0.94). It decreased for an increased duration of reduced working capacity above 30 days (compared to below 15 days) (Coefficient − 0.19, 95% CI -0.35- -0.03). We did not find a significant association between other EPTB manifestations and health utility estimates at the post-treatment stage.

**Table 5:** Multivariable regression analysis model presenting factors associated with the pre-treatment health utility per the EQ-5D-3L dimensions among lymphadenitis and pleuritis manifestations of Extrapulmonary Tuberculosis (EPTB)

EPTB manifestations	Factors associated with pre-treatment health utility among EPTB patients		Health Utility, mean (SD)	Univariable regression coefficient (95% Cl, *p*-value)*	Multivariable regression coefficient (95% Cl, *p*-value)*	
Lymphadenitis	Age-groups (years)	16–29	0.9 (0.1)	-	-	*n* = 122, Adjusted R-squared = 0.29
		30–44	0.9 (0.1)	0.00 (-0.05 to 0.06, *p* = 0.943)	0.02 (-0.03 to 0.07, *p* = 0.417)	
		Above 45	0.8 (0.3)	-0.10 (-0.17 to -0.03, *p* = 0.004)	-0.10 (-0.16 to -0.04, *p* = 0.003)	
	Health facilities visited for this illness	Dispensary	0.9 (0.1)	-	-	
		District Hospital	1.0 (0.1)	0.02 (-0.06 to 0.10, *p* = 0.602)	0.01 (-0.06 to 0.09, *p* = 0.727)	
		Health Center/Others	0.7 (0.6)	-0.27 (-0.39 to -0.14, *p* < 0.001)	-0.23 (-0.34 to -0.12, *p* < 0.001)	
		Private Hospital	0.9 (0.0)	0.01 (-0.16 to 0.18, *p* = 0.953)	-0.05 (-0.20 to 0.11, *p* = 0.561)	
		Regional Hospital	0.9 (0.1)	-0.00 (-0.05 to 0.04, *p* = 0.909)	-0.03 (-0.07 to 0.01, *p* = 0.180)	
	Percentage of reduced working capacity	Below 25%	1.0 (0.0)	-	-	
		25–50%	0.9 (0.1)	-0.04 (-0.11 to 0.02, *p* = 0.214)	-0.04 (-0.11 to 0.02, *p* = 0.177)	
		51–75%	0.9 (0.1)	-0.07 (-0.15 to 0.00, *p* = 0.065)	-0.08 (-0.15 to -0.00, *p* = 0.048)	
		76–100%	0.8 (0.4)	-0.23 (-0.33 to -0.12, *p* < 0.001)	-0.22 (-0.31 to -0.12, *p* < 0.001)	
Pleuritis	Socioeconomic status (SES)	Low SES	0.1 (0.6)	-	-	*n* = 39, Adjusted R-squared = 0.56
		Middle SES	0.8 (0.2)	0.65 (0.41 to 0.89, *p* < 0.001)	0.69 (0.46 to 0.92, *p* < 0.001)	
		High SES	0.8 (0.1)	0.70 (0.48 to 0.92, *p* < 0.001)	0.73 (0.52 to 0.94, *p* < 0.001)	
	Duration of reduced working capacity	Below 15 days	0.8 (0.2)	-	-	
		16–30 days	0.8 (0.3)	-0.06 (-0.25 to 0.12, *p* = 0.502)	-0.08 (-0.20 to 0.04, *p* = 0.203)	
		Above 30 days	0.7 (0.2)	-0.12 (-0.36 to 0.12, *p* = 0.320)	-0.19 (-0.35 to -0.03, *p* = 0.023)	

*CI = Confidence Interval

## Discussion

Our analyses showed that all EPTB manifestations affected five dimensions of HRQoL: physical (mobility, self-care, usual activities) and psychological (pain & discomfort and anxiety & depression). Notably, based on the EQ-5D-3L, the severity of EPTB illness across the reported manifestations varied. The frequency of EPTB Meningitis and pleuritis patients reporting health profiles portraying more severe disease was higher than that of lymphadenitis patients. A study of HRQoL among EPTB patients in Zanzibar also reported similar findings [6]. However, as we found, the physical and psychosocial impact of illness may vary with the EPTB manifestations. There were 1% of EPTB-lymphadenitis patients reporting extreme problems across all dimensions compared to none reported in Zanzibar. Moreover, the PROMs showed that the severe disease manifestations also had more pronounced psychosocial effects. Although the impact of psychosocial domains on the diagnosis and treatment of EPTB is known in TB high-burden settings like India and Nigeria [6], it remains understudied compared to the PTB [36, 37]. We found that pre- and post-treatment HRQoL was affected across all dimensions of health. These findings underscore the need for a comprehensive approach addressing psychosocial symptoms along with providing standard EPTB diagnosis and treatment. Therefore, EPTB disease manifestations and their implications should not be dealt with as a single entity.

The EuroQol HRQoL measuring instrument, such as EQ-5D-3L, allowed us to estimate a single measure of EPTB patients’ health utility. Such calculations can allow for comparing the impact of illness at different time points, patient groups, and diseases [24, 38]. For example, our estimates presented the effect of treatment in improving HRQoL. At the pre-treatment stage, the median health utility across EPTB manifestations ranged from 0.46 to 1.0. These values may be helpful during resource allocation or modulating policy by comparing with the health utility estimates of other diseases such as PTB (0.43 and 0.56) [39, 40]. Therefore, using generic instruments to record PROMs may have added value.

We found that the post-treatment HRQoL varied, and a sizeable proportion of patients across all the disease manifestations reported achieving the maximum level of health. However, we noted inequalities in reported health status among the various EPTB manifestations at the pre-treatment stage, although such inequalities moved towards improvement in health status following appropriate treatment. A higher proportion of patients with more severe EPTB manifestations, such as meningitis and pleuritis, showed a greater probability of improvement in HRQoL than lymphadenitis. This indicates that regardless of disease manifestation and severity, appropriate treatment alleviates HRQoL. Thus, if the presumptive EPTB patients are linked with a prompt diagnosis and treatment, their HRQoL would improve earlier. A similar response to ETPB treatment associated with HRQoL was reported in Zanzibar [6]. Compared to TB patients, at the post-treatment stage follow-up, a relatively higher proportion of non-TB patients reported extreme problems across five dimensions. A similar finding was also reported for PTB in India [41]. A systematic follow-up of non-TB patients was beyond the scope of this study. However, it again shows the need for a comprehensive health system approach, such as universal health coverage leading to timely diagnosis and appropriate treatment.

Using the estimated health utility as a summary measure to identify factors associated with EPTB HRQoL is novel. To our knowledge, this is the first study to calculate the health utility of EPTB using an EQ-5D questionnaire (Online Resource 7). Moreover, we reported findings categorised by EPTB manifestations. Our finding of lower health utility associated with access to primary health centres for EPTB treatment reflects patients’ preferences to attend public health sector services for less severe disease manifestations such as lymphadenitis. On the other hand, higher health utility for a relatively more severe disease manifestation, pleuritis, was associated with higher SES, illustrating the impact of patients’ affordability on their HRQoL. Such factors related to HRQoL may be non-modifiable from patients’ perspective unless prompt EPTB diagnostic and treatment facilities are available. Moreover, among the less severe manifestations (lymphadenitis), we found an association between health utility decrease and the extent of reduced working capacity. In contrast, the more severe manifestation (pleuritis) was associated with the duration of reduced working capacity. All these findings might show patients’ preference to access health systems based on disease severity, affordability and the impact of illness on working capacity. Thus, factors affecting the HRQoL of EPTB patients are embedded in access and utilisation of healthcare at the patient and health system levels, respectively.

This study had a few limitations. Our data was based on EQ-5D-3L instead of EQ-5D-5L, which appeared to have a ceiling effect. For example, despite presenting as presumptive EPTB patients at the pre-treatment stage, about 52% and 9% of the lymphadenitis and meningitis EPTB patients reported no problem across five dimensions of PROMs. If we had the data collection based on EQ-5D-5L, we might have found that such lymphadenitis or meningitis patients had few health problems not captured by EQ-5D-3L. We used the value set from Pakistan to estimate the health utility of our study participants from India. Although the patients’ socio-demographics of these two countries are close, a variation in PROMs for EPTB illness cannot be ruled out. Due to the limited available information about the HRQoL of EPTB patients, an extensive comparison with existing literature could not be done. We noted that three EPTB lymphadenitis patients reported worsening of their health states. We do not have available information to ascertain if this deterioration in health states is due to disease progression or related to potential side effects of TB treatment. Our follow-up of treatment ended with the completion of treatment. Therefore, we do not have information about the long-term persistence of problems in HRQoL, predominantly psychosocial, as they are known to recover relatively slowly [42]. Similarly, due to the long-term follow-up not included in our study design, TB treatment’s potential delayed side effects can be reported. We excluded patients whose PROMs were not available. Although their proportion is not large and we included patients above the estimated sample size, we cannot rule out if their information could sway the results of this study in another direction. There were no factors found to be associated with the non-TB patients’ HRQoL at the pre-treatment stage. Moreover, we were cautious about conducting such an analysis as we did not collect information about the non-TB patients’ diagnoses, which was beyond the scope of this study. This study analysis does not include clinical factors contributing to the improvement of HRQoL at the post-treatment stage. We did not have a systematic record of the non-TB diagnosis and treatment provided. This information could have allowed us to have more in-depth comparisons if available. Our study setting had a catchment area from rural settings. Although this may not affect the single measure of health utility estimates, the generalisability of results in terms of access and utilisation of healthcare should be carefully considered.

In conclusion, this study highlights that EPTB disease manifestations have varying impacts on different dimensions of HRQoL. Although studies assessing and improving HRQoL are not new, such assessments for EPTB have yet to be performed as part of the EPTB treatment and control initiatives. Therefore, to improve the health affected by EPTB, as we demonstrated, a holistic patient evaluation of all dimensions of health would be needed. Patients cannot cater for factors that reflect the influence of healthcare access and affordability based on SES. Considering our findings, multi-pronged interventions at patient and health system levels should be considered. For example, strengthening universal health coverage could be the optimal solution due to the range of signs and symptoms of EPTB. This would allow a prompt diagnosis and treatment despite patients’ interaction with the health system at any level. Any future studies should include additional clinical perspectives and assessment of factors contributing to improving HRQoL at the post-treatment stage. Such studies should also deploy the EQ-5D-5L questionnaire and a longer post-treatment follow-up of patients. This would allow the collection of more detailed data from patients’ perspectives and a delayed impact of illness or treatment, if any. Finally, despite the completion of the medical treatment, EPTB patients may still need support for gaps in their physical and psychosocial health. It is important that at the pre-treatment stage, in addition to the standard TB treatment, psychosocial support is provided to such patients as part of their treatment.

### Data sharing

The minimal dataset is included in the supplementary material (Online Resource 8), and additional information or any request for guidance can be sent to the corresponding author at shoaibraee@gmail.com. No patient-level identifiable data is included in this study.

## Electronic supplementary material

Below is the link to the electronic supplementary material.

Supplementary Material 1

Supplementary Material 2

Supplementary Material 3

Supplementary Material 4

Supplementary Material 5

Supplementary Material 6

Supplementary Material 7

Supplementary Material 8

## Data Availability

The minimal dataset is included in the supplementary material (Online Resource 8), and additional information or any request for guidance can be sent to the corresponding author at shoaibraee@gmail.com. No patient-level identifiable data is included in this study.
