# Supplementary material for: Health-related quality of life among extrapulmonary tuberculosis patients and inequalities by disease manifestations: a longitudinal study analysing the impact of TB treatment
Source: Qual Life Res. 2024 Dec 5;34(3):683–700. doi: 10.1007/s11136-024-03860-4 (PMC11920340; doi:10.1007/s11136-024-03860-4)
Supplement: Supplementary file 2 — Supplementary Material 2 [file 11136_2024_3860_MOESM2_ESM.docx]

**Online Resource 1**

**Article title:** Health-related quality of life among extrapulmonary tuberculosis patients and inequalities by disease manifestations: a longitudinal study analysing the impact of treatment.

**Journal name:** Quality of Life Research Journal

**Authors:** Shoaib Hassan*^1,2^, Manju Raj Purohit^3,4^, Mala Kanthali^3^, Reza Yaesoubi^2^, Swapnil Jain^5^, Tehmina Mustafa^1,6^

**Affiliations:**

1 Centre for International Health, Department of Global Public Health and Primary Care, University of Bergen, Bergen, Norway

2 Yale School of Public Health, Yale University, New Haven, USA

3 Department of Pathology, R.D. Gardi Medical College, Ujjain, India

4 Department of Public Health Sciences, Karolinska Institute, Stockholm, Sweden

5 Department of Respiratory Medicine, R.D. Gardi Medical College, Ujjain, India

6 Department of Thoracic Medicine, Haukeland University Hospital, Bergen, Norway

**Corresponding author:** Shoaib Hassan

**Email:** [shoaibraee@gamil.com](mailto:shoaibraee@gamil.com)

Patient flow diagram of presumptive extrapulmonary tuberculosis (EPTB) patients enrolled in the study analysis of the patient-reported outcome measures (PROMs).


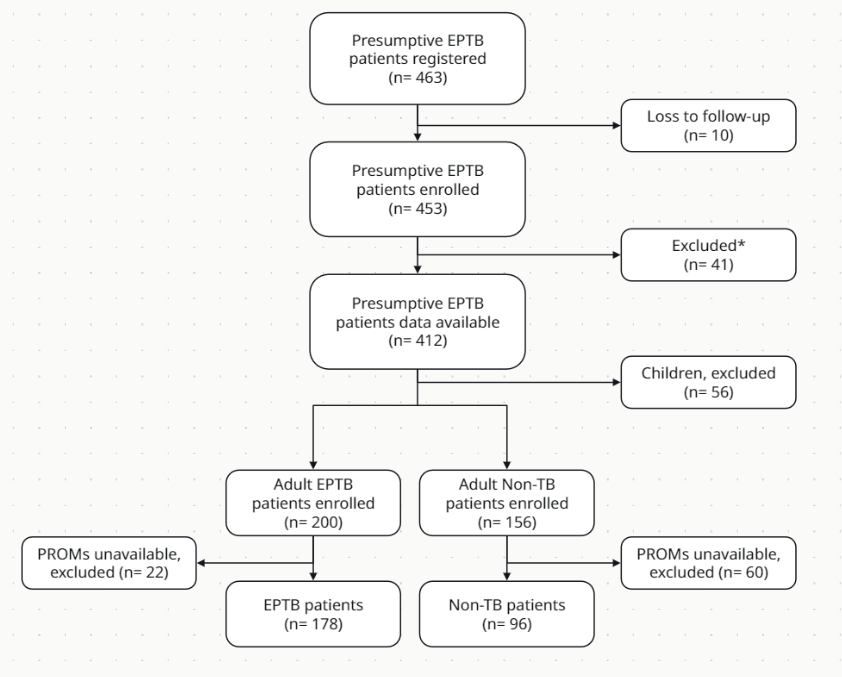


*Patients excluded per the study exclusion criteria: 39 patients who received TB treatment in the previous 12 months and 2 patients who did not provide informed consent.
