# Supplementary material for: Health-related quality of life among extrapulmonary tuberculosis patients and inequalities by disease manifestations: a longitudinal study analysing the impact of TB treatment
Source: Qual Life Res. 2024 Dec 5;34(3):683–700. doi: 10.1007/s11136-024-03860-4 (PMC11920340; doi:10.1007/s11136-024-03860-4)
Supplement: Supplementary file 3 — Supplementary Material 3 [file 11136_2024_3860_MOESM3_ESM.docx]

**Online Resource 2**

**Article title:** Health-related quality of life among extrapulmonary tuberculosis patients and inequalities by disease manifestations: a longitudinal study analysing the impact of treatment.

**Journal name:** Quality of Life Research Journal

**Authors:** Shoaib Hassan*^1,2^, Manju Raj Purohit^3,4^, Mala Kanthali^3^, Reza Yaesoubi^2^, Swapnil Jain^5^, Tehmina Mustafa^1,6^

**Affiliations:**

1 Centre for International Health, Department of Global Public Health and Primary Care, University of Bergen, Bergen, Norway

2 Yale School of Public Health, Yale University, New Haven, USA

3 Department of Pathology, R.D. Gardi Medical College, Ujjain, India

4 Department of Public Health Sciences, Karolinska Institute, Stockholm, Sweden

5 Department of Respiratory Medicine, R.D. Gardi Medical College, Ujjain, India

6 Department of Thoracic Medicine, Haukeland University Hospital, Bergen, Norway

**Corresponding author:** Shoaib Hassan

**Email:** [shoaibraee@gamil.com](mailto:shoaibraee@gamil.com)

Summary of EQ-5D-3L health states reported by Extrapulmonary tuberculosis (EPTB) and non-TB patients.

|  | | **EPTB patients** | **Non-TB patients** |
| --- | --- | --- | --- |
|  |  | No. State Freq. % Cumulative Cumulative  Freq % | No. State Freq. % Cumulative Cumulative  Freq % |
| **Lymphadenitis** | **Pre-treatment** | 1 11111 63 51.6 63 52 | 1 11111 57 71.2 57 71 |
|  |  | 2 22222 14 11.5 77 63 | 2 22222 7 8.8 64 80 |
|  |  | 3 11122 11 9.0 88 72 | 3 11122 2 2.5 66 83 |
|  |  | 4 11121 8 6.6 96 79 | 4 12222 2 2.5 68 85 |
|  |  | 5 11112 6 4.9 102 84 | 5 21122 2 2.5 70 88 |
|  |  | 6 22221 5 4.1 107 88 | 6 22221 2 2.5 72 90 |
|  |  | 7 12221 3 2.5 110 90 | 7 11112 1 1.2 73 91 |
|  |  | 8 12222 3 2.5 113 93 | 8 11121 1 1.2 74 93 |
|  |  | 9 12211 2 1.6 115 95 | 9 11222 1 1.2 75 94 |
|  |  | 10 11212 1 0.8 116 95 | 10 12221 1 1.2 76 95 |
|  |  | 11 12121 1 0.8 117 96 | 11 12232 1 1.2 77 96 |
|  |  | 12 12212 1 0.8 118 97 | 12 21222 1 1.2 78 98 |
|  |  | 13 21111 1 0.8 119 98 | 13 22211 1 1.2 79 99 |
|  |  | 14 21122 1 0.8 120 98 | 14 33322 1 1.2 80 100 |
|  |  | 15 22211 1 0.8 121 99 |  |
|  |  | 16 33333 1 0.8 122 100 |  |
|  | **Post-treatment** | 1 11111 109 89.3 109 89 | 1 11111 71 88.8 71 89 |
|  |  | 2 22222 5 4.1 114 93 | 2 22111 2 2.5 73 91 |
|  |  | 3 11211 3 2.5 117 96 | 3 22222 2 2.5 75 94 |
|  |  | 4 22211 2 1.6 119 98 | 4 33333 2 2.5 77 96 |
|  |  | 5 11122 1 0.8 120 98 | 5 11221 1 1.2 78 98 |
|  |  | 6 11221 1 0.8 121 99 | 6 22121 1 1.2 79 99 |
|  |  | 7 12211 1 0.8 122 100 | 7 23333 1 1.2 80 100 |
| **Pleuritis** | **Pre-treatment** | 1 22222 9 23.1 9 23 | 1 22222 2 33.3 2 33 |
|  |  | 2 11122 7 17.9 16 41 | 2 33333 2 33.3 4 67 |
|  |  | 3 12222 5 12.8 21 54 | 3 12222 1 16.7 5 83 |
|  |  | 4 11222 3 7.7 24 62 | 4 22221 1 16.7 6 100 |
|  |  | 5 11111 2 5.1 26 67 |  |
|  |  | 6 21222 2 5.1 28 72 |  |
|  |  | 7 22221 2 5.1 30 77 |  |
|  |  | 8 33333 2 5.1 32 82 |  |
|  |  | 9 11221 1 2.6 33 85 |  |
|  |  | 10 11333 1 2.6 34 87 |  |
|  |  | 11 12111 1 2.6 35 90 |  |
|  |  | 12 12122 1 2.6 36 92 |  |
|  |  | 13 21122 1 2.6 37 95 |  |
|  |  | 14 21232 1 2.6 38 97 |  |
|  |  | 15 32222 1 2.6 39 100 |  |
|  | **Post-treatment** | 1 11111 35 89.7 35 90 | 1 11111 4 66.7 4 66.7 |
|  |  | 2 11211 1 2.6 36 92 | 2 22222 2 33.3 6 100 |
|  |  | 3 12211 1 2.6 37 95 |  |
|  |  | 4 22211 1 2.6 38 97 |  |
|  |  | 5 22222 1 2.6 39 100 |  |
| **Meningitis** | **Pre-treatment** | 1 33333 5 45.5 5 46 | 1 22222 3 30 3 30 |
|  |  | 2 22222 3 27.3 8 73 | 2 33333 3 30 6 60 |
|  |  | 3 11111 1 9.1 9 82 | 3 22221 1 10 7 70 |
|  |  | 4 12211 1 9.1 10 91 | 4 22233 1 10 8 80 |
|  |  | 5 22233 1 9.1 11 100 | 5 32222 1 10 9 90 |
|  |  |  | 6 33332 1 10 10 100 |
|  | **Post-treatment** | 1 11111 5 45.5 5 46 | 1 11111 5 50 5 50 |
|  |  | 2 22222 2 18.2 7 64 | 2 22222 2 20 7 70 |
|  |  | 3 11122 1 9.1 8 73 | 3 11221 1 10 8 80 |
|  |  | 4 12111 1 9.1 9 82 | 4 22211 1 10 9 90 |
|  |  | 5 12211 1 9.1 10 91 | 5 33322 1 10 10 100 |
|  |  | 6 22221 1 9.1 11 100 |  |
| **Others** | **Pre-treatment** | 1 22222 2 33.3 2 33 |  |
|  |  | 2 11111 1 16.7 3 50 |  |
|  |  | 3 11112 1 16.7 4 67 |  |
|  |  | 4 13222 1 16.7 5 83 |  |
|  |  | 5 23322 1 16.7 6 100 |  |
|  | **Post-treatment** | 1 11111 4 66.7 4 67 |  |
|  |  | 2 21211 1 16.7 5 83 |  |
|  |  | 3 22211 1 16.7 6 100 |  |
