# Supplementary material for: Health-related quality of life among extrapulmonary tuberculosis patients and inequalities by disease manifestations: a longitudinal study analysing the impact of TB treatment
Source: Qual Life Res. 2024 Dec 5;34(3):683–700. doi: 10.1007/s11136-024-03860-4 (PMC11920340; doi:10.1007/s11136-024-03860-4)
Supplement: Supplementary file 4 — Supplementary Material 4 [file 11136_2024_3860_MOESM4_ESM.docx]

**Online Resource 3**

**Article title:** Health-related quality of life among extrapulmonary tuberculosis patients and inequalities by disease manifestations: a longitudinal study analysing the impact of treatment.

**Journal name:** Quality of Life Research Journal

**Authors:** Shoaib Hassan*^1,2^, Manju Raj Purohit^3,4^, Mala Kanthali^3^, Reza Yaesoubi^2^, Swapnil Jain^5^, Tehmina Mustafa^1,6^

**Affiliations:**

1 Centre for International Health, Department of Global Public Health and Primary Care, University of Bergen, Bergen, Norway

2 Yale School of Public Health, Yale University, New Haven, USA

3 Department of Pathology, R.D. Gardi Medical College, Ujjain, India

4 Department of Public Health Sciences, Karolinska Institute, Stockholm, Sweden

5 Department of Respiratory Medicine, R.D. Gardi Medical College, Ujjain, India

6 Department of Thoracic Medicine, Haukeland University Hospital, Bergen, Norway

**Corresponding author:** Shoaib Hassan

**Email:** [shoaibraee@gamil.com](mailto:shoaibraee@gamil.com)

The percentage of pre- and post-treatment health profiles reported by extrapulmonary tuberculosis patients summarised by the disease manifestation.

| **Lymphadenitis** | **Pre-treatment** | MO SC UA PD AD | |
| --- | --- | --- | --- |
|  |  | **1** 81.1 74.6 74.6 61.5 68.9 | |
|  |  | **2** 18.0 24.6 24.6 37.7 30.3 | |
|  |  | **3** 0.8 0.8 0.8 0.8 0.8 | |
|  | **Post-treatment** | MO SC UA PD AD | |
|  |  | **1** 94.3 93.4 90.2 94.3 95.1 | |
|  |  | **2** 5.7 6.6 9.8 5.7 4.9 | |
|  |  | **3** 0.0 0.0 0.0 0.0 0.0 | |
| **Pleuritis** | **Pre-treatment** | MO SC UA PD AD | |
|  |  | **1** 53.8 46.2 30.8 7.7 15.4 | |
|  |  | **2** 38.5 48.7 61.5 82.1 76.9 | |
|  |  | **3** 7.7 5.1 7.7 10.3 7.7 | |
|  | **Post-treatment** | MO SC UA PD AD | |
|  |  | **1** 94.9 92.3 89.7 97.4 97.4 | |
|  |  | **2** 5.1 7.7 10.3 2.6 2.6 | |
|  |  | **3** 0.0 0.0 0.0 0.0 0.0 |  |
| **Meningitis** | **Pre-treatment** | MO SC UA PD AD | |
|  |  | **1** 18.2 9.1 9.1 18.2 18.2 | |
|  |  | **2** 36.4 45.5 45.5 27.3 27.3 | |
|  |  | **3** 45.5 45.5 45.5 54.5 54.5 | |
|  | **Post-treatment** | MO SC UA PD AD | |
|  |  | **1** 72.7 54.5 63.6 63.6 72.7 | |
|  |  | **2** 27.3 45.5 36.4 36.4 27.3 | |
|  |  | **3** 0.0 0.0 0.0 0.0 0.0 |  |
| **Others** | **Pre-treatment** | MO SC UA PD AD | |
|  |  | **1** 50 33.3 33.3 33.3 16.7 | |
|  |  | **2** 50 33.3 50.0 66.7 83.3 | |
|  |  | **3** 0.0 33.3 16.7 0.0 0.0 |  |
|  | **Post-treatment** | MO SC UA PD AD | |
|  |  | **1** 66.7 83.3 66.7 100 100 | |
|  |  | **2** 33.3 16.7 33.3 0 0 | |
|  |  | **3** 0.0 0.0 0.0 0 0 |  |
