# Supplementary material for: Health-related quality of life among extrapulmonary tuberculosis patients and inequalities by disease manifestations: a longitudinal study analysing the impact of TB treatment
Source: Qual Life Res. 2024 Dec 5;34(3):683–700. doi: 10.1007/s11136-024-03860-4 (PMC11920340; doi:10.1007/s11136-024-03860-4)
Supplement: Supplementary file 6 — Supplementary Material 6 [file 11136_2024_3860_MOESM6_ESM.docx]

**Online Resource 5**

**Article title:** Health-related quality of life among extrapulmonary tuberculosis patients and inequalities by disease manifestations: a longitudinal study analysing the impact of treatment.

**Journal name:** Quality of Life Research Journal

**Authors:** Shoaib Hassan*^1,2^, Manju Raj Purohit^3,4^, Mala Kanthali^3^, Reza Yaesoubi^2^, Swapnil Jain^5^, Tehmina Mustafa^1,6^

**Affiliations:**

1 Centre for International Health, Department of Global Public Health and Primary Care, University of Bergen, Bergen, Norway

2 Yale School of Public Health, Yale University, New Haven, USA

3 Department of Pathology, R.D. Gardi Medical College, Ujjain, India

4 Department of Public Health Sciences, Karolinska Institute, Stockholm, Sweden

5 Department of Respiratory Medicine, R.D. Gardi Medical College, Ujjain, India

6 Department of Thoracic Medicine, Haukeland University Hospital, Bergen, Norway

**Corresponding author:** Shoaib Hassan

**Email:** [shoaibraee@gamil.com](mailto:shoaibraee@gamil.com)

Multivariable regression analysis model presenting demographic, socioeconomic and clinical factors associated with the pre-treatment health utility per the EQ-5D-3L dimensions among Extrapulmonary tuberculosis (EPTB) patients.

(n=178, Adjusted R^2^= 0.30)

| Factors associated with pre-treatment health utility among EPTB patients | | Mean  (SD) | Univariable regression coefficient  (95% Cl, p-value)* | Multivariable regression coefficient  (95% Cl, p-value)* |
| --- | --- | --- | --- | --- |
| Gender | Female | 0.9 (0.2) | - | - |
|  | Male | 0.8 (0.3) | -0.09 (-0.17 to -0.02, p=0.017) | 0.02 (-0.14 to 0.19, p=0.804) |
| Age-groups (years) | 16-29 | 0.9 (0.2) | - | - |
|  | 30-44 | 0.9 (0.3) | -0.03 (-0.12 to 0.06, p=0.523) | -0.04 (-0.14 to 0.05, p=0.334) |
|  | Above 45 | 0.8 (0.3) | -0.12 (-0.21 to -0.02, p=0.021) | -0.12 (-0.27 to 0.03, p=0.120) |
| Education levels | Primary or below | 0.8 (0.3) | - | - |
|  | Middle or Secondary | 0.9 (0.2) | 0.05 (-0.03 to 0.13, p=0.248) | -0.04 (-0.13 to 0.05, p=0.344) |
|  | Higher | 0.9 (0.1) | 0.10 (-0.01 to 0.21, p=0.082) | 0.01 (-0.12 to 0.14, p=0.876) |
| Marital status | Married | 0.9 (0.2) | - | - |
|  | Unmarried | 0.9 (0.2) | 0.02 (-0.06 to 0.09, p=0.619) | 0.00 (-0.13 to 0.13, p=0.989) |
| Socioeconomic status (SES) | Low SES | 0.8 (0.4) | - | - |
|  | Middle SES | 0.9 (0.2) | 0.12 (0.00 to 0.23, p=0.045) | 0.06 (-0.06 to 0.18, p=0.301) |
|  | High SES | 0.8 (0.3) | 0.06 (-0.06 to 0.17, p=0.324) | 0.01 (-0.12 to 0.15, p=0.829) |
| Occupation | Govt Employed | 0.9 (0.2) | - | - |
|  | Housewife | 0.9 (0.2) | -0.01 (-0.12 to 0.10, p=0.841) | -0.00 (-0.14 to 0.14, p=0.992) |
|  | Unemployed | 0.8 (0.3) | -0.08 (-0.20 to 0.03, p=0.158) | -0.05 (-0.21 to 0.11, p=0.552) |
|  | Private Employed | 0.8 (0.3) | -0.07 (-0.19 to 0.04, p=0.226) | 0.05 (-0.11 to 0.21, p=0.546) |
| Previous history of TB | No | 0.9 (0.2) | - | - |
|  | Yes | 0.8 (0.3) | -0.03 (-0.15 to 0.08, p=0.593) | 0.04 (-0.08 to 0.16, p=0.541) |
| Family history of TB | No | 0.9 (0.3) | - | - |
|  | Yes | 0.8 (0.2) | -0.02 (-0.12 to 0.07, p=0.645) | 0.04 (-0.06 to 0.14, p=0.446) |
| Self-medication | No | 0.9 (0.3) | - | - |
|  | Yes | 0.9 (0.2) | -0.01 (-0.10 to 0.09, p=0.905) | -0.06 (-0.16 to 0.05, p=0.279) |
| Hospitalisation status | Inpatient | 0.7 (0.3) | - | - |
|  | Outpatient | 0.9 (0.1) | 0.19 (0.12 to 0.26, p<0.001) | -0.01 (-0.09 to 0.08, p=0.904) |
| Patient-level delay | Above Median PD | 0.9 (0.2) | - | - |
|  | Below Median PD | 0.8 (0.3) | -0.03 (-0.10 to 0.05, p=0.442) | -0.00 (-0.09 to 0.08, p=0.912) |
| Health system level delay (before study site visit) | Above Median SD | 0.9 (0.2) | - | - |
|  | Below Median SD | 0.9 (0.3) | 0.01 (-0.06 to 0.08, p=0.796) | 0.01 (-0.10 to 0.11, p=0.871) |
| Health system level delay (after study site visit) | Below Median SD | 0.9 (0.2) | - | - |
|  | Above Median SD | 0.8 (0.3) | -0.07 (-0.14 to 0.01, p=0.085) | -0.08 (-0.16 to -0.00, p=0.044) |
| Duration of fever | [0.0,730.0] | 0.9 (0.3) | -0.00 (-0.00 to 0.00, p=0.224) | 0.00 (-0.00 to 0.00, p=0.779) |
| Duration of weight loss | [0.0,730.0] | 0.9 (0.3) | -0.00 (-0.00 to 0.00, p=0.136) | 0.00 (-0.00 to 0.00, p=0.381) |
| Duration of appetite loss | [0.0,730.0] | 0.9 (0.3) | -0.00 (-0.00 to 0.00, p=0.240) | -0.00 (-0.00 to 0.00, p=0.641) |
| Duration of night sweats | [0.0,730.0] | 0.9 (0.3) | -0.00 (-0.00 to -0.00, p=0.036) | -0.00 (-0.00 to -0.00, p=0.012) |
| Duration of fatigue | [0.0,730.0] | 0.9 (0.3) | -0.00 (-0.00 to 0.00, p=0.585) | 0.00 (-0.00 to 0.00, p=0.100) |
| Duration of amenorrhea | [0.0,365.0] | 0.9 (0.3) | -0.00 (-0.00 to -0.00, p=0.006) | -0.00 (-0.00 to 0.00, p=0.392) |
| Duration of body weakness | [0.0,1825.0] | 0.9 (0.3) | -0.00 (-0.00 to 0.00, p=0.454) | -0.00 (-0.00 to 0.00, p=0.298) |
| Duration of frequent colds | [0.0,365.0] | 0.9 (0.3) | -0.00 (-0.00 to 0.00, p=0.463) | 0.00 (-0.00 to 0.00, p=0.556) |
| Duration of neck mass | [0.0,1460.0] | 0.9 (0.3) | 0.00 (-0.00 to 0.00, p=0.157) | 0.00 (-0.00 to 0.00, p=0.336) |
| Number of health facilities visited for this illness | One | 0.9 (0.2) | - | - |
|  | Two | 0.8 (0.3) | -0.03 (-0.12 to 0.06, p=0.496) | 0.01 (-0.10 to 0.13, p=0.805) |
|  | Three | 0.8 (0.4) | -0.06 (-0.24 to 0.12, p=0.508) | 0.18 (-0.09 to 0.44, p=0.182) |
|  | Missing | 0.9 (0.2) | 0.03 (-0.07 to 0.13, p=0.531) | 0.04 (-0.23 to 0.31, p=0.788) |
| Number of visits to health facilities for this illness | One | 0.9 (0.1) | - | - |
|  | Two | 0.8 (0.3) | -0.07 (-0.16 to 0.02, p=0.153) | -0.03 (-0.15 to 0.08, p=0.575) |
|  | Three | 0.8 (0.3) | -0.11 (-0.26 to 0.03, p=0.128) | -0.17 (-0.39 to 0.05, p=0.118) |
|  | Four | 0.6 (0.5) | -0.25 (-0.48 to -0.02, p=0.031) | -0.21 (-0.53 to 0.10, p=0.186) |
|  | Missing | 0.9 (0.2) | -0.02 (-0.12 to 0.09, p=0.776) | -0.03 (-0.28 to 0.21, p=0.789) |
| Time to reach the nearest health facility | Below 30 min | 0.9 (0.1) | - | - |
|  | Between 30- 60 min | 0.9 (0.3) | -0.05 (-0.14 to 0.03, p=0.208) | 0.03 (-0.05 to 0.12, p=0.429) |
|  | Above 60 min | 0.8 (0.3) | -0.16 (-0.25 to -0.07, p=0.001) | -0.03 (-0.15 to 0.10, p=0.680) |
| Time to reach this health facility (study site) | 30- 60 min | 0.8 (0.3) | - | - |
|  | Above 60 min | 0.8 (0.3) | -0.00 (-0.11 to 0.10, p=0.938) | -0.01 (-0.12 to 0.10, p=0.821) |
|  | Below 30 min | 0.9 (0.2) | 0.07 (-0.02 to 0.16, p=0.135) | -0.02 (-0.11 to 0.07, p=0.607) |
| Travel and wait time to this health facility (study site) | Below 10 min | 0.9 (0.1) | - | - |
|  | 10- 60 min | 0.9 (0.2) | -0.05 (-0.14 to 0.05, p=0.315) | -0.05 (-0.15 to 0.05, p=0.332) |
|  | 60- 120 min | 0.7 (0.4) | -0.20 (-0.29 to -0.10, p<0.001) | 0.01 (-0.15 to 0.17, p=0.927) |
|  | Above 120 min | 0.7 (0.4) | -0.27 (-0.40 to -0.14, p<0.001) | -0.03 (-0.22 to 0.16, p=0.739) |
| Health facilities visited for this illness | Dispensary | 0.9 (0.2) | - | - |
|  | District Hospital | 0.9 (0.3) | -0.01 (-0.14 to 0.12, p=0.856) | -0.07 (-0.22 to 0.08, p=0.365) |
|  | Health Center/Others | 0.7 (0.5) | -0.18 (-0.41 to 0.05, p=0.123) | -0.09 (-0.28 to 0.11, p=0.374) |
|  | Private Hospital | 0.8 (0.3) | -0.12 (-0.41 to 0.17, p=0.431) | -0.32 (-0.63 to -0.02, p=0.037) |
|  | Regional Hospital | 0.9 (0.2) | -0.02 (-0.10 to 0.06, p=0.587) | -0.09 (-0.20 to 0.02, p=0.096) |
| Duration of reduced working capacity | Below 15 days | 0.9 (0.2) | - | - |
|  | 16- 30 days | 0.8 (0.3) | -0.09 (-0.17 to -0.00, p=0.041) | 0.01 (-0.08 to 0.10, p=0.797) |
|  | Above 30 days | 0.9 (0.1) | -0.00 (-0.10 to 0.09, p=0.948) | 0.01 (-0.08 to 0.10, p=0.793) |
| Percentage of reduced working capacity | Below 25% | 1.0 (0.1) | - | - |
|  | 25- 50% | 0.9 (0.1) | -0.03 (-0.14 to 0.09, p=0.616) | -0.07 (-0.18 to 0.03, p=0.176) |
|  | 51- 75% | 0.9 (0.1) | -0.09 (-0.22 to 0.03, p=0.151) | -0.12 (-0.25 to 0.01, p=0.080) |
|  | 76- 100% | 0.6 (0.4) | -0.37 (-0.50 to -0.24, p<0.001) | -0.14 (-0.29 to 0.01, p=0.062) |

*CI= Confidence Interval
